# Supplementary material for: A Smartphone-Based Whole-Cell Array Sensor for Detection of Antibiotics in Milk
Source: Sensors (Basel). 2019 Sep 9;19(18):3882. doi: 10.3390/s19183882 (PMC6767005; doi:10.3390/s19183882)
Supplement: Supplementary file 1 [file sensors-19-03882-s001.pdf]

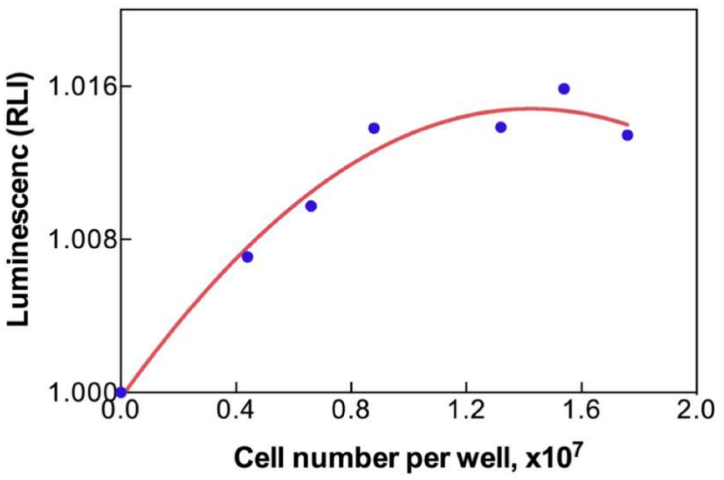

Figure S1. Optimization of bacteria cell density for LCS detection. Luminescence from different concentrations of bacteria, ranging from 8 × 10^8 to 3.2 × 10^9 CFU/mL (4.4 × 10^6 to 1.76 × 10^7 cells per well), stimulated with 16 ng/mL of CIP for 180 min.

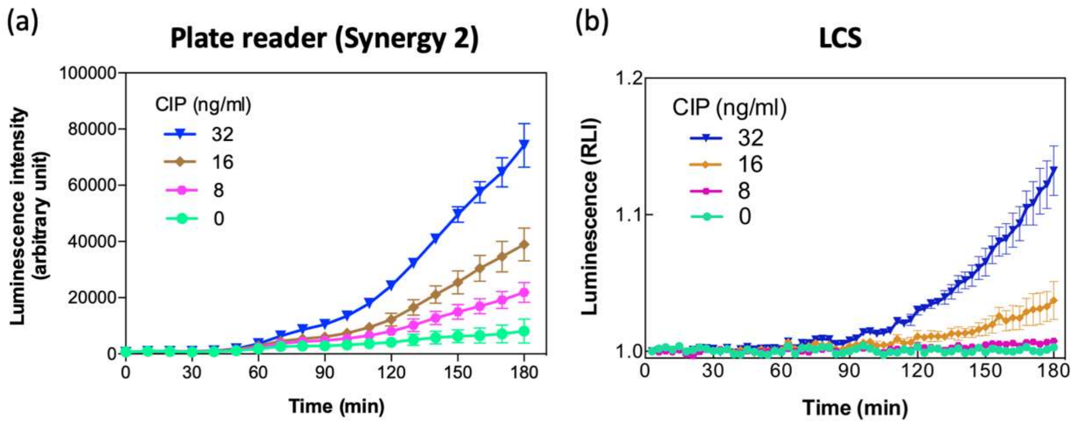

Figure S2. Luminescence of immobilized bacteria (1.32 x 10^7 cells per well of 96-well plate) stimulated with 0, 8, 16, or 32 ng/mL of CIP in LB was determined by commercially plate reader Synergy 2 (**a**) or the LCS system (**b**).

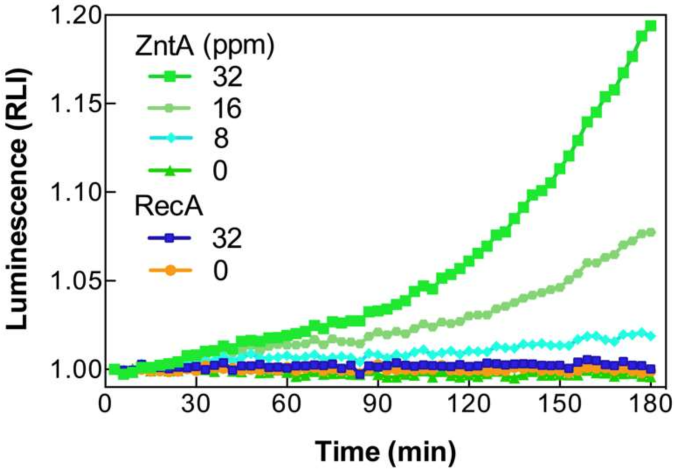

Figure S3. Time-lapse luminescence of immobilized bacteria (1.32 × 10^7 cells per well) stimulated with 0, 8, 16, 32 ppm of cadmium chloride. ZntA: bacteria harboring *ZntA promoter::luxCDABE*; RecA: bacteria harboring *RecA promoter::luxCDABE*.

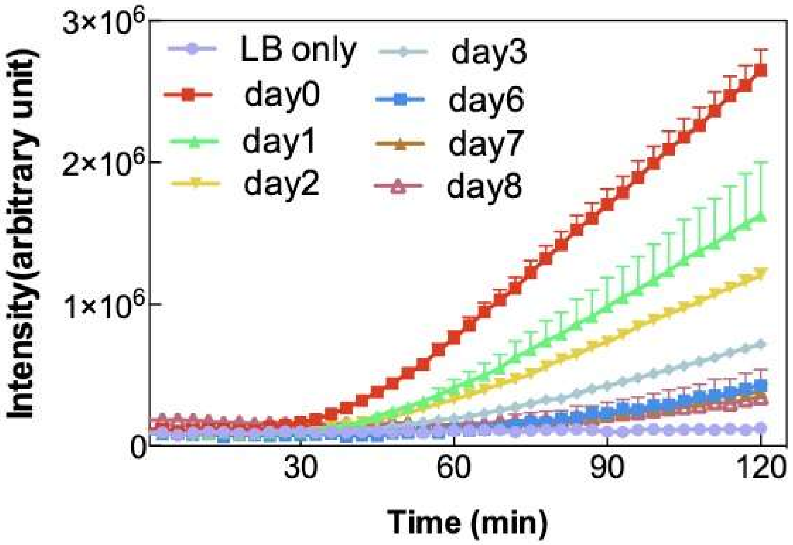

Figure S4. Time-lapse luminescence of immobilized bacteria in chip stored at 4 °C for the designated time. The test has been carried out in our previous LumiSense system.
